# Evaluation of a novel CD20-targeted IL-15 immunotherapeutic with potent activity against B cell lymphoma

**DOI:** 10.1186/2051-1426-2-S3-P122

**Published:** 2014-11-06

**Authors:** Bai Liu, Lin Kong, Warren D Marcus, Xiaoyue Chen, Kaiping Han, Peter R Rhode, Hing C Wong

**Affiliations:** 1 Altor BioScience Corporation, Miramar, FL, USA

## 

IL-15 exhibits potent antitumor efficacy in mouse models through its ability to promote proliferation and activation of NK cells and memory CD8^+ ^T cells. Recently, targeting approaches have been developed to direct these activities against tumor cells and minimize potential toxicities related to systemic immune activation. IL-15 and its receptor α (IL-15Rα) are co-expressed in antigen presenting cells allowing trans-presentation of IL-15 to immune effector cells. We previously reported that the high-affinity interactions between an IL-15 superagonist (IL-15N72D) and the extracellular IL-15Rα sushi domain (IL-15RαSu) could be exploited to create a functional scaffold for the design of multivalent disease-targeted protein complexes. Extending these findings to relevant tumor antigens, a tetravalent complex (2B8T2M) was generated comprising the single-chain anti-human CD20 Fv domain of Rituximab linked to the N-termini of the IL-15N72D and IL-15RαSu-Fc fusion proteins. As designed, this complex was found to retained IL-15 activity to induce proliferative and effector responses of CD8^+ ^T cells and NK cells as well as CD20- and Fc receptor-binding activity necessary to mediate ADCC and CDC against CD20-positive B cell lymphoma. Surprisingly, 2B8T2M (≥1 nM) was also capable of inducing significant apoptosis of B cell lymphoma cells, which was not observed following incubation with Rituximab (Daudi cell death: 2B8T2M; 36.5 ± 0.5% vs. Rituximab; 8.5 ± 0.3%). Moreover, treatment of tumor-bearing SCID mice with 2B8T2M (12.5 mg/kg, day 15, 18 post-tumor injection) was more effective than Rituximab (10 mg/kg equivalent to 12.5 mg/kg 2B8T2M) in reducing levels of CD20-positive Daudi lymphoma cells in the bone marrow (% BM Daudi cells at day 22: 2B8T2M, 1.4 ± 1.3% vs. Rituximab, 28.1 ± 6.2%; vs. PBS, 42.1 ± 8.0%; both p < 0.01). This antitumor activity was dependent on each of the three binding domains of 2B8T2M. In addition, 5 mg/kg 2B8T2M treatment of Daudi tumor-bearing SCID mice significantly prolonged survival compared to PBS control- and 10 mg/kg Rituximab-treatment groups (median survival: 2B8T2M, 42 days vs. Rituximab, 35 days; vs. PBS, 27 days; both p < 0.01). In cynomolgus monkeys, 2B8T2M administration (5 mg/kg, day 0, 3) also exhibited greater activity than Rituximab (10 mg/kg) for depleting B cells in the blood and lymph nodes (p < 0.05). 2B8T2M treatment was well tolerated in each of these models. Together, these finding demonstrate that tumor antigen-targeted IL-15 complexes can stimulate and direct immune responses to more effectively eliminate tumor cells than related therapeutic antibodies. Thus, these molecules represent novel and promising targeted immunotherapeutics for treating cancer.

## Acknowledgements

NIH/NCI grant CA174091 (Wong).

